# Acute Kidney Injury: Global Health Alert

**DOI:** 10.1016/j.hkjn.2013.03.001

**Published:** 2013-02-01

**Authors:** P. K. Tao Li, E. A. Burdmann, R. L. Mehta

**Affiliations:** 1 *Department of Medicine and Therapeutics, Prince of Wales Hospital, Chinese University of Hong Kong, Hong Kong*; 2 *Department of Medicine, University of Sao Paulo Medical School, Sao Paulo, SP, Brazil*; 3 *Department of Medicine, University of California San Diego, San Diego, CA, USA*

**Keywords:** Acute Kidney Injury, Health, Morbidity, Mortality, Prevention and control

## Abstract

Acute kidney injury (AKI) is increasingly prevalent in developing and developed countries and is associated with severe morbidity and mortality. Most etiologies of AKI can be prevented by interventions at the individual, community, regional and in-hospital levels. Effective measures must include community-wide efforts to increase an awareness of the devastating effects of AKI and provide guidance on preventive strategies, as well as early recognition and management. Efforts should be focused on minimizing causes of AKI, increasing awareness of the importance of serial measurements of serum creatinine in high risk patients, and documenting urine volume in acutely ill people to achieve early diagnosis; there is as yet no definitive role for alternative biomarkers. Protocols need to be developed to systematically manage prerenal conditions and specific infections. More accurate data about the true incidence and clinical impact of AKI will help to raise the importance of the disease in the community, increase awareness of AKI by governments, the public, general and family physicians and other health care professionals to help prevent the disease. Prevention is the key to avoid the heavy burden of mortality and morbidity associated with AKI.

## INTRODUCTION TO WORLD KIDNEY DAY 2013

On March 14, 2013, the 8^th ^World Kidney Day (WKD) will be celebrated. WKD is an annual event jointly organized by the International Society of Nephrology and the International Federation of Kidney Foundations. This year, we aim to alert the public to the global increase in acute kidney injury (AKI) in both developing and developed countries. AKI is a syndrome of abrupt loss of kidney function, often with oliguria, which is strongly associated with increased early and long-term patient morbidity and mortality, as well as the subsequent development of chronic kidney disease (CKD).

There is an urgent need for a global health strategy to reduce the enormous growing burden of AKI and its consequences. We advocate that efforts focused on preventing AKI be coupled with early detection and treatment, and adequate follow up to reduce mortality and the long-term burden of AKI-induced CKD.

## EPIDEMIOLOGY OF AKI WORLDWIDE

The KDIGO (Kidney Disease Improving Global Outcome) Clinical Practice Guideline for AKI, defines AKI as any of the following: increase in serum creatinine by ≥0.3 mg/dL (≥26.5 µmol/L) within 48 hours; or increase in serum creatinine to ≥1.5 times baseline, which is known or presumed to have occurred within the prior seven days; or urine volume <0.5 mL/kg/h for 6 hours [1]. An epidemiological study in Scotland showed that the incidence of AKI was 2,147 per million population per year (pmp) [2] and in a community study in northern California, the annual incidence of non-dialysis requiring and dialysis requiring AKI were respectively 3841 and 244 pmp [3]; this incidence increased over time and was consistently higher in men and in the elderly [3]. Unfortunately, there are still no comprehensive studies on the incidence of AKI in the community in the developing world.

Recent hospital studies in the developed world report AKI in 3.2%–9.6% of admissions, with overall in-hospital mortality around 20%, and up to 50% in ICU patients [4, 5]. There is also increased long-term mortality in those with AKI surviving hospitalization, with adjusted mortality risk of 1.4, which augmented with increasing severity of AKI [5]. AKI requiring renal replacement therapy occurs in 5%–6% of ICU patients, with an extremely high in-hospital mortality rate of 60% [6]. It is estimated that about two million people die of AKI every year [6.^7^]. Those who survive AKI have a higher risk for later development of CKD [8].

## AKI IN THE DEVELOPING WORLD

Eighty-six percent of the world’s population lives in low- and middle-income countries, which have many contrasts and inequalities. Sophisticated tertiary hospitals co-exist with inadequate primary care and poor health system infrastructure in the same country and even in the same city. In such countries, AKI has a peculiar bimodal presentation. In modern, large, urban centers, the pattern of AKI is very similar to that found in the developed world; it is predominantly a hospital-acquired disease occurring mostly in older, critically ill multiorgan failure patients with substantial comorbidity. The main cause for AKI in this population is renal ischemia, principally due to sepsis, and often associated with nephrotoxic drugs [9].

At the same time, in rural areas or smaller cities in the countryside, AKI will usually be a community-acquired disease, affecting younger and previously healthy individuals. In this population specific causes of AKI include diarrheal diseases with dehydration, infectious diseases (malaria, dengue, yellow fever, leptospirosis, tetanus and human immunodeficiency virus), animal venoms (snakes, bees, *Loxosceles* spiders, *Lonomia caterpillars*), septic abortion, dyes and natural medicines [10-12]. Most of these factors triggering AKI are associated with poverty and affect vulnerable populations because of poor sanitation and water hygiene (diarrheal diseases), a lack of education and access to an adequate urban infrastructure and difficulty to have access to the health care system (septic abortions, snakebite, natural medicines, tetanus) and breaking of an ecological balance from uncontrolled and unplanned urbanization (leptospirosis, yellow fever, Africanized bees and *Lonomia caterpillar* accidents) [10-13]. In the developing world, the same ICU may have a typical bacterial sepsis-induced AKI patient side-by-side with a patient suffering from dengue or tetanus-induced AKI.

Increasingly these causes of AKI may be exported from developing to developed countries due to immigration, business travel, tourism and world warming.

## AKI IN THE DEVELOPED WORLD

The availability of standardized criteria for diagnosis and staging of AKI has made clear that the prevalence of AKI in the developed world has increased in the last decade [14, 15]. AKI is now encountered in 45% of patients admitted to the ICU and 20% of hospitalized patients [16, 17]. This increased prevalence likely reflects an aging population burdened by multiple co-morbidities, which is often managed with multiple drugs [18, 19]. AKI is a multifactorial entity. Etiological factors include prerenal injury contributing to reduced renal perfusion, however the precipitating events are often iatrogenic, *e.g.*, hypotension during anesthesia and surgery, or profound diarrhea secondary to *C. difficile* infection resulting from aggressive antibiotic therapy [20]. Drug-induced kidney injury is recognized as a major factor in about 20% of cases, while hospital-acquired infections, sepsis, complex surgery and diagnostic procedures requiring intravenous contrast continue to be significant risk factors for development of AKI [21-23]. Patients in the ICU are dying of AKI and not just simply with AKI. Experimental and small observational studies have shown that AKI negatively affects immunity and is associated with higher rates of infection [24]. AKI patients frequently develop a vicious cycle of immune dysfunction, sepsis and multiorgan failure. Indeed, severe sepsis is currently the major cause of AKI in the US [25]. The host response to sepsis involves an inflammatory response which activates innate immunity. If this persists, the immune response will lead to a release of a multitude of proinflammatory products, which frequently cause organ dysfunction, including kidney failure [26].

A key issue in the developed world is that patients are increasingly cared for by multiple providers, often in different health care systems, with infrequent or minimal data sharing between providers and across health care systems. This lack of knowledge often results in overdosing of nephrotoxic medications, for example a dentist might prescribe large doses of non-steroidal anti-inflammatory drugs (NSAIDs) for pain management after dental surgery without the knowledge of a patient’s underlying CKD, thus contributing to development of AKI superimposed on the CKD. Since kidney disease is generally silent, unless it is severe enough to reduce urine output or lead to complications, it can often go unrecognized [27]. A recent national audit of the care provided to patients who died with a diagnosis of AKI in the UK hospitals revealed several shortcomings. AKI was often diagnosed late in the course, the initial severity was underestimated, and diagnostic and therapeutic interventions were often incomplete or delayed [28]. This audit illustrates the urgent need for improving awareness of AKI and has prompted the medical community in the UK to implement specific measures to facilitate early recognition, timely diagnosis, and appropriate management and follow-up of AKI patients [29].

## AKI IN CHILDREN

The epidemiology of pediatric AKI has shifted in the last decades from intrinsic kidney diseases such as hemolytic uremic syndrome and glomerulonephritis to ischemia, nephrotoxins and sepsis in critically ill children [17]. Estimates of the incidence of AKI in children vary depending on the definition used and the population assessed, but it is clearly increasing. A pediatric-modified RIFLE (Risk, Injury, Failure, Loss, and End-stage kidney) criterion was developed and validated in 2007. The major difference with the original RIFLE definition is the use of changes in estimated creatinine clearance calculated by Schwartz formula rather than serum creatinine, in view of the large variation in body mass in children [17]. Development of AKI has been consistently demonstrated as an independent risk factor for death in children, from neonates to adolescents. Recently, the concept of “renal angina” was proposed as a tool to the early identification of kidney injury together with early adoption of preventive measures in children at high risk for development of AKI [30]. One of the strongest indicators of renal angina and risk of further development of AKI in children is fluid overload [17, 30]. As in adults, AKI carries a significant risk for late development of CKD in surviving children [17, 31].

## OTHER CONSEQUENCES OF AKI

Apart from the high mortality associated with AKI, there are other major consequences. Patients with AKI utilize more resources and have longer hospital lengths of stay in part due to effect of AKI on other organ function. For instance, AKI patients have more difficulty being weaned off ventilators [32]. AKI patients are more prone to fluid overload with a resultant increase in mortality and impaired renal recovery [33]. When patients leave hospital they generally require prolonged recuperation often in skilled nursing facilities and may not recover kidney function [34]. In a study of over 4000 type 2 diabetic patients in the Veterans Affairs health care system in the US, approximately half required one or more hospitalizations, and among those requiring hospitalization 29% experienced at least one episode of AKI [35]. CKD is now recognized as a major non-communicable disease, and data in the same study of type 2 diabetics showed that AKI was an important independent risk factor for stage 4 CKD (hazard ratio 3.56), with each AKI episode doubling that risk. There is other consistent and increasing evidence that AKI contributes to CKD development and may result in dialysis dependency [8, 36]. Collectively these data demonstrate the high personal and community costs of an episode of AKI and stress the pressing need to address this problem in an effective way [37]. 

## IS AKI PREVENTABLE AND TREATABLE?

A central tenet of the WKD message since 2006 has been that “kidney disease is common, harmful and treatable.” Like CKD, AKI is common, harmful and treatable, and is also largely preventable.

The heterogeneity of patients and the broad range of situations where AKI is encountered make it challenging to standardize an approach for evaluating and managing patients with this syndrome. The recent KDIGO guidelines for management of AKI provide a useful reference to assist clinicians for managing AKI, however the successful implementation of guidelines and their application to individual patients can be slow and requires concerted efforts [1, 38].

Prevention of AKI starts in the community with prompt assessment of those at risk, for example in taking prompt action following effective evaluation of the severity of fluid depletion in acute diarrhea. Regular drug therapy can compound that risk and the many older people taking NSAIDs or renin-angiotensin system blockers should be educated to discontinue them temporarily in the face of acute intercurrent illness, the so-called “medication holiday.”

In the developed world, the growing adoption of electronic medical records (EMRs) provides several opportunities for managing patients through the continuum of outpatient and in-hospital care. Several studies have now shown that active surveillance for changes in creatinine can automate alerts to guide drug dosing and reduce the incidence of drug-induced kidney injury [39, 40]. An “AKI sniffer system” embedded in the EMR to warn physicians of changing renal function has been shown to increase the number and timeliness of early therapeutic interventions [41]. The emerging field of kidney specific biomarkers of damage will additionally offer opportunities to improve care [42]. Several studies have now shown the ability of various biomarkers alone or in combination to facilitate earlier diagnosis and improve differential diagnosis of AKI. However, biomarker-guided interventions have not as yet been shown to be of benefit [43], and currently serum creatinine and urinary volume remain the clinical pointers to AKI diagnosis. Given advances in medical informatics, biomarker development and interpretation, and therapeutic interventions, it is now imperative that we leverage these advances to educate physicians and care providers about AKI and provide them with the tools to manage these patients timely and effectively.

In the hospital setting, AKI preventive measures continue to be adequate hemodynamic control, hydration, hematocrit and oxygen profiling, and avoidance of nephrotoxic drugs; other preventive maneuvers should be implemented for particular diseases or conditions causing AKI. In the developing world, awareness of the specific infectious or venomous organisms in certain areas will allow environmental protection, vaccines, pharmacologic prophylaxis, and early administration of anti-venom. Early and adequate anti-venom administration is a valuable preventive maneuver for snakebite and caterpillar venom induced AKI, reducing its morbidity and lethality [10, 13]. Prompt diagnosis, timely hemodialysis and adequate supportive therapy are associated with improved outcome in tropical infectious disease-associated AKI, such as leptospirosis and malaria [10, 44, 45]. As always, the early diagnosis of AKI is the key to minimize further insults.

Prevention for AKI is clearly the key to avoid the heavy burden of mortality and morbidity associated with this syndrome (Table 1), and this will only come about through increasing awareness of the true incidence and clinical impact of AKI among governments, the public, general and family physicians and other health care professionals. Most etiologies of AKI can be prevented by interventions at the individual, community, regional and in-hospital levels. Effective measures must include community-wide efforts to increase an awareness of the devastating effects of this illness and provide guidance on preventive strategies and for early recognition and management. Efforts should be focused on minimizing AKI causes, increasing awareness of the importance of serial measurements of serum creatinine in high risk patients and observing urinary volume to achieve early diagnosis. Protocols need to be developed to systematically manage prerenal conditions and specific infections.

**Table 1:** Strategies for preventing AKI

Government	Funding support for AKI research in hospital and community on AKI incidence, outcome and mortality
	Funding support for setting up AKI registries
	Recognition of natural hazards for AKI: water sanitation, flooding, venomous animals
	Recognition of AKI in common infections: malaria, dengue, leptospirosis, HIV, post-infectious hemolytic uremic syndrome
	Better obstetric care
	Collaboration with health care professionals on educating the public about AKI prevention
Public	Aware of the potential problems of AKI and avoid unsupervised, indiscriminate and long-term use of nephrotoxic drugs and natural substances
General practitioners and physicians	Awareness of patients at risk for AKI and situations contributing to AKI
	Aware of pre-renal causes of AKI and of the need for early and appropriate rehydration and hemodynamic optimization in hypovolemic patients
	Aware of natural and man-made nephrotoxin, nephrotoxic drugs, herbs and indigenous medicine
	Judicious use of nephrotoxic drugs and aware of potential drug interactions
	Early recognition of AKI and early referral to nephrologists
Nephrologists	Establish and implement common AKI diagnostic criteria and definitions for prevention, treatment and research
	Find new diagnostic tools including inexpensive technology and biomarkers for AKI diagnosis and monitoring
	Adapt renal replacement therapy to regional needs, technique and resource availability

## RENAL REPLACEMENT THERAPY FOR AKI

When AKI patients require renal replacement therapy (RRT), the current KDIGO recommendations are to deliver an effluent volume of 20–25 mL/kg/h for continuous renal replacement therapy (CRRT) or to deliver a Kt/V of 3.9 per week when using intermittent or extended RRT [1]. This requires careful monitoring as there is often a significant reduction in the dose delivered *vs* that prescribed [46]. Peritoneal dialysis (PD) should also be considered for AKI, particularly in developing countries, because it is a simple, effective, safe and relatively inexpensive form of RRT [47]. The technical simplicity of PD and the potential to reduce costs if consumables can be made locally, present an opportunity to establish cost-effective programs for managing AKI as has been shown in a recently established PD program for managing AKI in one of the poorest countries in Africa [48].

## CONCLUSION

The worldwide celebration of World Kidney Day 2013 provides an opportunity to share the message that acute kidney injury is indeed common, harmful, preventable and treatable, and that protecting the kidneys from this lethal syndrome is an important health strategy for the patient and the community. The effective implementation of such strategies will only come when both the general public and the renal community work together to convince health authorities of the pressing need to do this. Government and health authorities must allocate resources to manage this problem both in the developed and developing world.
